# Three models of community mental health services In low-income countries

**DOI:** 10.1186/1752-4458-5-3

**Published:** 2011-01-25

**Authors:** Alex Cohen, Julian Eaton, Birgit Radtke, Christina George, Bro Victor Manuel, Mary De Silva, Vikram Patel

**Affiliations:** 1 Faculty of Epidemiology & Population Health, London School of Hygiene & Tropical Medicine, Keppel Street, London WC1E 7HT, UK; 2 CBM National Co-ordination Office,13 Okemesi Crescent, Garki 2, Abuja, Nigeria; 3 Community Mental Health, CBM, Nibelungenstr. 124, 64625 Bensheim, Germany; 4 Department of Psychiatry, Dr Somervell Memorial CSI Medical College, Karakonam, Thiruvananthapuram, Kerala, India; 5 Holy Face Rehabilitation Centre for Mental Health, Tabaco City, Albay Province, the Philippines

## Abstract

**Objective:**

To compare and contrast three models of community mental health services in low-income settings.

**Data Sources/Study Setting:**

Primary and secondary data collected before, during, and after site visits to mental health programs in Nigeria, the Philippines, and India.

**Study Design:**

Qualitative case study methodology.

**Data Collection:**

Data were collected through interviews and observations during site visits to the programs, as well as from reviews of documentary evidence.

**Principal Findings:**

A set of narrative topics and program indicators were used to compare and contrast three community mental health programs in low-income countries. This allowed us to identify a diversity of service delivery models, common challenges, and the strengths and weaknesses of each program. More definitive evaluations will require the establishment of data collection methods and information systems that provide data about the clinical and social outcomes of clients, as well as their use of services.

**Conclusions:**

Community mental health programs in low-income countries face a number of challenges. Using a case study methodology developed for this purpose, it is possible to compare programs and begin to assess the effectiveness of diverse service delivery models.

## Introduction

Global Mental Health has emerged as a significant field in public health [1], as evidenced by series in *The Lancet *[2-7] and *PLoS Medicine *[8-14], as well as by the establishment of the WHO mental health Gap Action Programme [15] and the development of intervention guidelines for the treatment of mental, neurological and substance use disorders in non-specialized settings [16]. However, a major obstacle to the goal of improving and expanding mental health services in low-income countries (LIC) is the comparative lack of evidence about how mental health services in LIC function in actual practice [17]. The Case Studies Project at the London School of Hygiene and Tropical Medicine was established with the objective of developing a methodology to address this deficiency. In this paper, we compare and contrast three models of community mental health services in low-income settings, based on information gathered according to a qualitative case study methodology that we have developed.

## Methods

### Settings

The three programs that are the foci of this paper are:

• Services for People with Disabilities, Abuja, Federal Capital Territory, Nigeria;

• Holy Face Rehabilitation Center for Mental Health, Tabaco City, Albay Province, the Philippines;

• Asia Psychosocial Rehabilitation Program, Karakonam, Kerala, India.

The programs that were to be the subjects of the case studies were selected by the CBM Mental Health Advisory Working Group. All of the programs described here are funded, in part, by CBM, an international non-governmental organization that funds programs for persons with disabilities in many of the world's poorest countries.

### Design

A qualitative case study methodology was employed to document the services of the three programs. The major component of the study was site visits (by AC) of five to ten days to each program: November 2008 and January 2009 (Nigeria), June 2009 (the Philippines), and November 2009 (India). Site visit activities included accompanying staff in the field and on home visits, observing community clinics, visiting with Self-Help Groups, and interviewing staff. Whenever possible, documentary evidence was utilized to confirm and/or augment the information collected during site visits.

### Measures

Information about the programs was collected according to a set of narrative topics and program-level indicators (Table 1) that were developed and refined during the course of the study.

**Table 1:** Narrative topics, program-level indicators and outcome measures

Narrative topics	• Context and history of the program • Present status • Program management • Nature of interventions ○ Clinical ○ Psychosocial • Engagement with political systems • Health systems in which program functions
**Program-level indicators**	• Use of protocols and guidelines • Medication supplies • Transportation • Accessibility of services • Case-finding methods • Treatment coverage • Self-Help Groups & Livelihood Programs • Referral systems • Information systems • Program costs

**Outcome measures**	• Symptom severity • Functional status • Quality of life

### Data collection

Observations, information about the programs and interviews with key personnel were documented by AC in narrative accounts of site visits. When possible, interviews were audio-recorded and then transcribed. Program records were reviewed to collect statistical data. Information was also derived from documents, e.g. brochures, materials on the internet, annual reports, and periodic site visit reports by CBM staff. After the site visits, AC often asked (via email) program and CBM staff to fill in gaps in the information already collected.

### Analysis

Following the site visits, AC converted the narrative accounts, transcripts of interviews, and, whenever possible, the documentary evidence, into text files that were analyzed (HyperResearch, version 2.8.3) according to the narrative topics and program-level indicators. Site visit reports were then prepared by AC and, to be certain that the reports were accurate and did not contain significant gaps, circulated among the heads of the programs, the CBM Mental Health Advisory Working Group, and the co-authors at LSHTM. To present the results here, we have arranged information about the programs into the following domains: History and context; Pathways to care and characteristics of clients; Treatment coverage; Accessibility of services; Clinical interventions; Psychosocial Interventions; Referral pathways; Outcomes.

## Results

### History, context, and organization

The three programs discussed here all began operations in the period 2004-2006 when CBM piloted a number of community mental health programs in order to assess their feasibility and effectiveness when carried out as components of Community-Based Rehabilitation projects in LIC. The program in Abuja, Services for People with Disabilities, was established because several CBM staff recognized the need for mental health care - mainly for psychosis and epilepsy - in Nigeria. The Holy Face Rehabilitation Center for Mental Health, in Tabaco City, the Philippines, was established, first, as a result of discussions between CBM and the Brothers of Charity - a non-governmental organization with a focus on care for persons with mental disorders - and, second, because a Bishop in the Bicol Region of the Philippines appealed to the Brothers to provide care for mentally ill indigents. The Asia Psychosocial Rehabilitation Program evolved out of the crisis brought about by the December 2004 Indian Ocean tsunami that devastated communities on the southwestern coast of India in the states of Kerala and Tamil Nadu.

The Abuja program operates in the Federal Capital Territory of Nigeria, which encompasses the central city of Abuja, a number of satellite towns, and large rural areas with dispersed populations. The Catholic Archdiocese of Abuja operates and partially funds the program. CBM provides about half the funding. The long-term goal is to have the Archdiocese support the program entirely, with CBM providing expertise and occasional financial assistance. Mental health services are just one component of Services for People With Disabilities, a Community-Based Rehabilitation program that provides services for a range of physical disabilities, as well as psychosocial interventions to promote economic integration and social inclusion. The program employs 17 field workers who are each based in a different zone of the Federal Capital Territory. The field workers are responsible for case-finding and follow-up of all clients, not just those receiving mental health services. One community psychiatric nurse is responsible for the clinical care of all mental health clients (Table 2).

**Table 2:** Three models of community mental health services in low-resource settings

	Services for People With Disabilities	Holy Face Rehabilitation Center for Mental Health	Asia Psychosocial Rehabilitation Program
HISTORY & CONTEXT			
Established	2006	2004	2005
**Location**	Abuja, Federal Capital Territory, Nigeria	Tabaco City, Albay Province, Philippines	Dr Somerville Memorial CSI Medical College & Hospital, Karakonam, Kerala India
			Community catchment areas: Pozhiyoor, Thiruvananthapuram, Kerala Thuthoor & Colachel, Kanyakumari,Tamil Nadu

**Geographic area**	8,000 sq km [29]	Albay Province: 2,553 sq km	Kerala: 25 sq km*
		Bicol Region: 18,130 sq km [30]	Kanyakumari: 60 sq km

**Population**	1.4 million[29]	Albay Province: 1.3 million Bicol Region: 5.7 million [30]	100,000

**Socioeconomic Context**	Low-income, but within a region of great economic inequalities	Low-income	Mostly low-income

**Human Development Index**	.511 (Nigeria) [31]	.577 (Albay Province) [32]	.763 (Kanyakumari, TN) [34]
			.775 (Kerala) [33]

**ORGANIZATION**			
**Model**	Mental health services comprise one component of a multi-faceted CBR program	Residential facility and outpatient clinic; on-going collaborations with 2 CBR programs and local governments	Inpatient and outpatient clinics at a general hospital; community-based activities and clinics; collaboration with CBR program

**Funding**	• Archdiocese of Abuja • CBM • Internally generated revenue from drug receipts	• Brothers of Charity • CBM • Municipalities of Tiwi and Tabaco City subsidize client expenses and provide transportation to outpatient clinic.	• CBM • Dr Somerville Memorial CSI Medical College & Hospital^†^

**Management**	Director Administrative Assistant	Director 2 Administrative staff	Director Project Officer 1 Program Coordinator

**Staff**	Full-time 17 FWs 1 CPN 1 Physio assistant 1 Special Educationalist 1 driver Part-time 1 consulting psychiatrist^‡^	Full-time 2 social workers 3 nurses 1 field worker Part-time 1 psychiatrist Non-clinical staff 6 Brothers of Charity; 2 security guards; 1 driver; 1 maintenance person; 1 cook; 1 laundry person	Full-time 2 social workers Part-time 2 psychiatrists^§^ 1 psychologist^§^ 1 social worker^§^ 10 CVs

	**Services for People With Disabilities**	**Holy Face Rehabilitation Center for Mental Health**	**Asia Psychosocial Rehabilitation Program**

**PATHWAYS TO CARE**			
Case-finding	Active presence of FWs in the community; community clinics. Enrolled 76 new clients (2008): 80% epilepsy, 20% psychosis.	No direct outreach. Have trained community health workers who, in turn, refer clients. 354 new clients (2008); 119 new clients (first six months of 2009). Data (2007) suggest ≈80% of new clients have schizophrenia, remainder appear to have major depressive disorder.	2009: House-to-house surveys in catchment areas (≈2,000 house visits) Cases detected = 181 • CMD = 94 • Psychosis = 55 • Other = 32 New cases recruited = 60 • CMD = 33 • Psychosis = 13 • Other = 14

Outreach	FWs and CPN will hold periodic meetings in local churches; FWs will consult with parish priests and village chiefs	Social workers and nurses will hold periodic community workshops and mental health trainings for local government health workers and NGO community volunteers	All staff participate in community meetings; consultations with parish priests

Follow-up	Home visits by FWs; follow-up treatment during home visits by CPN or community clinics	Every week, FW looks for clients who have not returned to outpatient clinic; presence of local government and NGO health workers in the community. Follow-up treatment in weekly outpatient clinic.	Home visits by CVs; follow-up treatment in community clinics and/or at the hospital

**CLIENT CHARACTERISTICS**			
**Number**	≈88 per month (2008)**	354 new clients (2008) 119 new clients (Jan-May 2009)^††^ Since 2004,120 admitted to residential facility & about 1,800 seen as outpatients (as of May 2009) 70 to 120 patients are seen in the outpatient clinic each week Inpatient facility never has more than 20 residents.	2009: Colachel: 90 received services, and, of these, 25 received inpatient treatment and followed-up in the community. 30 active clients as of December. Pozhiyoor: 35 clients received services; of these, 5 received inpatient treatment. Thuthoor and surrounding areas: 42 clients received services. Of these, 22 accessed care first at the hospital and subsequently followed in the community. TOTAL = 167

**Clinical characteristics**	Epilepsy (≈75%) & psychosis	Psychosis & CMD	CMD (≈50%) Psychosis (≈25%) Other disorders (≈25%)

**INTERVENTIONS**			
**Clinical interventions**	Older psychotropic medications	Older psychotropic medications	Full range of psychotropic medications; specialist psychotherapy and non-specialist counseling

	**Services for People With Disabilities**	**Holy Face Rehabilitation Center for Mental Health**	**Asia Psychosocial Rehabilitation Program**

**Psychosocial interventions**	SHGs (3)	FSGs; livelihood activities Supporting FSGs in 4 municipalities and 1 island in Albay Province (as of June 2009)	SHGs; livelihood activities; prevention and promotion groups; community workshops 14 SHGs with a total of 204 members, of whom 36 (almost 18%) were clients and caregivers (Nov 2009).

* Does not include areas added in 2010-2011

^† ^Dr Somervell Memorial CSI Medical College & Hospital provides a portion of staff salaries, as well as administrative and infrastructure support. Patients are eligible to buy insurance that is subsidized by the college. This will partially cover costs of the program.

^‡ ^CBM Mental Health Advisor, supporting many similar projects, all of which receive occasional support visits.

^§ ^These staff are based at the hospital and have duties that are not part of the program.

** Estimated from number of client visits in the year

^†† ^No information on number of active clients

The Tabaco City program was established as a residential facility for people with chronic psychoses and an outpatient clinic for individuals suffering from a range of mental disorders. The program receives funding from the Brothers of Charity and, through the end of 2010 received support from CBM Australia, which is presently reviewing a Tabaco City program proposal for multi-year support. Two local municipalities - Tiwi and Tabaco City - provide limited funding for clients in the residential program and for the purchase of medications for outpatients. The inpatient and outpatient facilities are located about 12 km from the center of Tabaco City, on 10 acres of a former coconut plantation at the foot of Mt. Mayon, an active volcano. The staff consists of two social workers, three nurses, one psychiatrist (who works one day a week), and one field worker who have a degree in sociology. The program also employs administrative and house keeping staff. In addition, several of the resident Brothers of Charity conduct classes for the residential clients (Table 2).

The Karakonam program is based in the Department of Psychiatry at Dr. Somervell Memorial CSI Medical College & Hospital. The program is funded by CBM, but salaries for staff are paid, in part, by the hospital, which also provides the infrastructure for the inpatient and outpatient facilities and makes it possible for the program to offer inpatient care for people with acute psychiatric episodes and serious medical conditions. The hospital's catchment area covers the Trivandrum and Kanyakumari districts of Kerala and Tamil Nadu, respectively, but the mental health program covers a much smaller areas. The program maintains offices in the communities of Colachel, Pozhiyoor, and Thuthoor, and offers monthly clinics in the first two, while clients in the third must travel to the hospital for treatment. Clients in Colachel and Pozhiyoor can also, if they choose, to seek treatment at the hospital. The program employs two psychiatrists (part-time in the community, the remainder in the hospital), one part-time psychologist, three social workers, and 10 community volunteers (Table 2).

Salaries for staff in all of the programs are comparatively low, particularly for the lay mental health workers in the Abuja and Karakonam programs.

The programs all function in regions with high levels of poverty. According to the Human Development Index, a composite measure of life expectancy, education levels, and standard of living, the areas in which the Karakonam program functions appear to be better off than Albay Province and Nigeria as a whole (Table 2).

Health services, in general, are lacking in Abuja, and virtually nonexistent for neuropsychiatric disorders. The Karakonam program operates within a complex health system of private, non-profit, government, and Ayurvedic services - although the available practitioners are not sufficiently trained to treat mental disorders effectively. The Tabaco City program operates in a setting somewhere in-between, although Albay Province, with its hospitals (e.g., the Bicol Medical Center, which includes a psychiatric facility) more closely resembles the southwest coastal region of India than Abuja. Seeking the help of traditional or alternative healers is common in Nigeria and India; anecdotal evidence suggests that this is not true in Albay Province. In regard to mental health services, specifically, precise data are not available for the program settings. However, data from the WHO Project Atlas [18] indicate that mental health resources are extremely limited in Nigeria, India, and the Philippines.

Because of the size of the catchment areas, or in the case of the Karakonam program the need to travel one to two hours from the hospital to reach the communities being served, provision of transportation for staff is critical. The Abuja program provides field workers with motorbikes. The program also owns a pick-up truck that is used to transport the community psychiatric nurse and the psychiatrist when conducting field visits. The Tabaco City program mostly relies on public transportation for staff to travel to meetings in the community. Staff are reimbursed for these costs. Hospital-based staff in the Karakonam program are driven to the catchment areas by a car service that is associated with the hospital. A motorbike was given to one of the social workers so that he could travel among the catchment areas. The community volunteers walk or take public transportation in the course of their fieldwork or to attend meetings at the hospital. The program reimburses them for their transportation costs.

### Pathways to care & characteristics of clients

The Abuja program field workers and the Karakonam program community volunteers conduct active case-finding. This entails going house-to-house in search of potential clients and consulting key informants, e.g., village chiefs or parish priests. However, there are major differences in the approaches to case-finding. First, the Abuja program field workers are responsible for locating persons with mental disorders and persons with physical disabilities, while the Karakonam program community volunteers are only trying to locate persons with mental disorders. Second, since the Karakonam program was established in response to the tsunami, it was originally oriented to identifying persons suffering from common mental disorders. In contrast, the mental health component of the Abuja program was established because of the burden of epilepsy and psychosis in Nigeria, and the recognition that treatments for these disorders would be feasible and effective in the context of a community-based rehabilitation program (Table 2).

The Tabaco City program does not conduct case-finding and depends on referrals from local government health workers and two non-governmental organizations that provide community rehabilitation services in Albay Province (Table 2).

All three programs conduct out-reach activities, e.g., consultations with government officials, other non-governmental organizations, and community leaders, as well as holding public meetings to inform people about the services offered. The Abuja program trains members of the community to recognize and refer individuals to the local field worker. The Tabaco City program trains municipal and non-governmental health workers to refer individuals and to follow-up current clients. Soon after it started, the Karakonam program trained school teachers to recognize and refer children who were displaying behavioral problems (Table 2).

One can also assume that former or current clients act as informal sources of referral by suggesting to friends or family members that they seek help for a mental disorder by visiting an Abuja program field worker in his office, going to the weekly outpatient clinic at the Tabaco City program, or attending one of the monthly community clinics provided by the Karakonam program.

Even though the programs all provide multiple pathways to care, the clinical characteristics of clients in the three programs are markedly different (Table 2). This is surprising given that the programs operate in settings that offer few, if any, mental health services and one would expect that individuals with a broad range of disorders would seek and receive care. Yet, a large majority of clients in Abuja receive treatment for epilepsy while the large majority of clients in the Tabaco City program receive treatment for psychosis. In contrast, about half of the clients in the Karakonam program are being treated for a common mental disorder and only one-third are being treated for psychosis.

### Treatment coverage

As can be seen in Table 2, the geographic and population sizes of the catchment areas in Nigeria and the Philippines are very large. Yet, the number of clients receiving care from the Abuja and Tabaco City programs indicates that the programs are reaching only a tiny fraction of those in need of care. An estimate of the prevalence of psychosis (3.3%) [19] suggests that there may be as many as 43,000 persons with psychosis in Albay Province, and an estimate of the prevalence of epilepsy in sub-Saharan Africa (1.5%) [20] suggests that there are about 21,000 persons with epilepsy in Abuja. The Karakonam program serves much smaller catchment areas. Assuming that about 10% of a given population will be suffering from a mental disorder at any given time [21], one can estimate that about 10,000 persons are in need of mental health services.

Together, these data suggest that, according to the available epidemiological evidence, the three programs are reaching only a tiny fraction of potential clients. Furthermore, one might speculate that large geographic or political boundaries are not necessarily accurate reflections of actual service areas. For example, the Bicol Region is the stated catchment area of the Tabaco City program, but a review of records indicates that the vast majority of clients come from Albay Province. In the case of the Abuja and the Karakonam programs, determining the effective composition of the mental health service areas will require further research, i.e., mapping where clients reside.

### Accessibility of services

Accessing services does not appear to be an issue for clients of the Abuja and Karakonam programs. The former provides most treatment in the homes of clients, whereas the latter provides services in the community and at the hospital, which is readily accessible via public transportation. However, the small number of clients in the Abuja program leads one to speculate that barriers to care do exist. Further research is required to identify those barriers.

Even though the Tabaco City program provides services to the largest number of clients, its distance from city center and the expense of transportation are barriers to care. We were told that some families could not afford the expense of transportation to the facility. As a result, clients stopped attending the program's day hospital services when a free daily van service was discontinued because of budget cuts. Budget cuts also forced the program to discontinue its van service to the weekly outpatient clinic. Fortunately, Tabaco City now operates the van service and the outpatient clinic continues in operation.

The Abuja program operates a drug revolving fund that sells medications at a small fraction above cost, which is generally affordable to clients and families. CBM also maintains a fund to help those who cannot afford the medications. Anecdotal reports suggest that the cost of medication to clients in the Tabaco City program, even though it is sold on a sliding scale, leads to a degree of non-adherence. No doubt, the cost of outpatient sessions (between $2.25 and $4.50) also makes services inaccessible for some clients.

The Karakonam program, using its own funds, provides medication free of charge to clients. This is possible, at least in part, because medications are comparatively inexpensive in India.

### Clinical Interventions

The Abuja program community psychiatric nurse is responsible for the diagnosis and medication management of all mental health clients. Clinical care takes place during home visits or at a clinic in a remote rural community. Psychiatrists in the Tabaco City and Karakonam programs provide clinical care during outpatient or community clinics (Table 2).

Medication is the mainstay of treatment in all three programs. Limited funding means that the Abuja and Tabaco City programs must rely on older, less expensive medications. An additional challenge for the Abuja program is ensuring the quality of medications since much of what is available in Nigeria is of poor quality [22]. Therefore, the program purchases, whenever possible, medications from the Christian Health Association of Nigeria, which imports high-quality generic medications. Only the Karakonam program has ready access to newer medications, including atypical antipsychotics and newer anti-depressants. None of the programs have developed explicit guidelines and protocols for the use of medication. All programs regularly inquire about side effects.

Unlike the other programs, the Karakonam program has specialists who have the time and expertise to provide formal psychotherapy.

The Tabaco City and Karakonam programs offer inpatient services. However, the former limits admissions to chronic patients who do not require acute care, while the latter provides care in a hospital with extensive resources and is capable of caring for persons who are acutely ill.

None of the three programs are well-equipped to handle emergencies in the community, although the Karakonam program is in the best position to mobilize resources if necessary.

The Abuja and Karakonam programs follow-up clients regularly. The Abuja program community psychiatric nurse tries to see clients once a month. During these visits, the nurse will, if necessary dispense medications. Field workers attend these visits and will visit clients at other times, too. The Karakonam community volunteers visit clients twice a month. Clients mostly receive their medications in the community clinics or at the hospital, although community volunteers do make home deliveries of medications to about 10% of the clients. The Tabaco City program relies, for the most part, on municipal and non-governmental organization community health workers to follow-up clients.

### Psychosocial interventions

All of the programs have organized Self-Help Groups, referred to as Family-Support Groups in the Tabaco City program.

The Tabaco City program has organized Family Support Groups in several communities of Albay Province (Table 2). One advantage of membership, at least for the group in Tabaco City, is that members are eligible to participate in two Livelihood Programs: skills training and loans to raise pigs or to establish or expand small roadside shops. Meetings of the groups are structured and include psychoeducation, sharing of experiences, and updates on livelihoods. Anecdotal reports suggest that membership in Family Support Groups is associated with fewer relapses and more regular clinic attendance of clients.

The Karakonam program offers the broadest range of psychosocial interventions. Follow-up sessions with community volunteers may include interventions aimed at improving self-care, activities of daily living, and social functioning. Community volunteers also provide informal counseling, e.g., listening, relaxation techniques, and support for caregivers. Additionally, community volunteers organize and run on-going promotion and prevention activities for groups of children (6-15 years), adolescents (16-19 years), and adults. Topics discussed in the groups include nutrition, hygiene, study skills, stress management, substance abuse, mental illness, and (for with the adults) communicable and chronic illnesses. The program also has organized Self-Help Groups for women, which, in addition to providing social support, undertake income-generating activities, e.g., soap making, tailoring, selling cloth with printed designs, and operating small shops. Unlike the groups organized by the other programs, the groups organized by the Karakonam program are open to all women, and, thus, may provide clients and care-givers greater opportunities for social integration.

The Abuja program recently started one Self-Help Group in each of three zones. Plans are in place to eventually organize groups in all of the zones. The intention of the groups is to foster advocacy, provide peer support, and participate in livelihood activities.

### Referral pathways

The absence of psychiatric services in the Federal Capital Territory makes it difficult for the Abuja program to refer cases for specialist services - this is only possible if a family can bring the client to a better-resourced city. Clients can be referred to other components of the community-based rehabilitation program, e.g., the economic integration program, but such referrals are not made frequently. The Tabaco City program has not established official referral pathways with a nearby medical center and a psychiatric hospital. In contrast, because the Karakonam program is based at a hospital that also maintains community primary care centers, referring clients for treatment of physical disorders or inpatient psychiatric care is done routinely.

### Outcomes

Although all of the programs collect data, none organize it so that statistics are readily available on the number of clients receiving services or clients' basic clinical and sociodemographic characteristics. Nor can the programs provide data about the treatments that clients are receiving, the length of time clients have been in treatment, or the consistency with which clients have accessed services. Last, data on the outcomes of intervention are not available. However, this is changing. The Karakonam program has (as of September 2009) initiated monitoring of clinical, functional, and caregiver outcomes, as well as client satisfaction. Additionally, the Abuja program is currently piloting a data collection system that will enable the generation of routine process and outcome data.

## Discussion

This paper describes the results of case studies of three community mental health programs in LIC. Information was collected according to a set of narrative topics and program-level indicators, then analyzed and organized to describe, compare, and contrast the programs. In this way, we found a diversity of service delivery models, common challenges, and unique strengths and weaknesses of each program. Furthermore, the case study methodology that we developed was found to be a useful tool to examine the strategies used by community mental health programs to deliver services within the constraints of low-resource settings.

Two common challenges were found. Above all, without CBM funding it is unlikely that any of the programs would continue to exist, at least in their present form. Without CBM funding and technical expertise the Catholic Archdiocese of Abuja would likely reduce the scope of the Abuja program, and there is no guarantee that the mental health services would be retained. Without funding from CBM the Brothers of Charity would, no doubt, continue to support the Tabaco City program but would probably be forced to curtail or discontinue the community programs and the weekly outpatient clinic. The Karakonam program might survive without CBM funding, but only if the Dr Somerville Memorial CSI Medical College & Hospital managed to secure other sources of support.

The second challenge is the comparative lack of human resources. In Abuja, there are no psychiatrists to which the program community psychiatric nurse can refer challenging cases. For the Tabaco City program the situation is different: there are psychiatrists in Albay Province but, at least when the program was established, it was difficult to find one who would accept the position. In contrast, the Karakonam program has been successful in recruiting qualified psychiatrists and social workers - probably because it is based at a teaching hospital - but there is always the danger that they will be lured away by higher salaries in the United Kingdom, North America, or Australia. It is also difficult to retain female community volunteers because after getting married they often stop working. Hiring men as community volunteers is not easy since men can make much more money working as fishermen.

Despite the challenges, the programs all have strengths. Most importantly, all manage to function and deliver services to persons who, without those services, would likely be neglected. In addition, program staff must be given credit for their support and respect for clients, and the ability to remain positive and dedicated even though their work is physically and emotionally difficult, pays poorly, and offers little chance for advancement within any of the programs.

Nevertheless, one must not ignore obvious weaknesses. For example, despite the size of its target population, the Abuja program has provided services to relatively few clients. The program could also be criticized for its lack of specialist support for the community psychiatric nurse. Two weaknesses can be readily identified in the Tabaco City program. First, its clinical staff is not large enough to handle the number of residential or outpatient clients. Second, its relatively isolated location means that it is difficult to consider it a community-based program. The Karakonam program is well-resourced with specialists who are willing to work in community settings, knowledgeable and skilled community volunteers, a wide range of medications, and access to a large hospital. At the same time, this means it is not a likely candidate as a model for most low-income settings.

The programs described here represent a range of service models [23]: from an exclusively community-based model (the Abuja program), to one (the Tabaco City program) that is mostly clinic-based but with some activities in the community, to a model that provides community, clinic, and hospital care (the Karakonam program). Each of these models may be appropriate, depending on the available human, technical, and financial resources in a given setting. It is critical, however, when planning or reforming services, that developers in other settings consider the strengths and weaknesses of each of these programs and models.

The evidence presented here also suggests that when planning or establishing programs it is critical to consider whether program resources are reasonably sufficient for providing services in the intended catchment area. For example, the Abuja program community psychiatric nurse must travel many hours and long distances to visit clients in each of the 18 zones of the Federal Capital Territory, and the Tabaco City program psychiatrist must see 70-120 clients during each outpatient clinic. Both situations demand tremendous efforts. Whether these efforts are sustainable and/or undermine the quality of services is a question that must be examined.

A major strength of the case study methodology that we have developed is its ability to provide detailed descriptions of models of care and how they function within specific sociocultural and socioeconomic contexts. Using a defined set of narrative topics and program-level indicators also allows one to compare and contrast the programs, whether they utilize the same or different models. A knowledge base of case studies would be an invaluable resource in attempts to scale up mental health service in LIC.

The methodology that was the basis for our case studies has several limitations. First, most of the evidence was collected during relatively short site visits. Longer visits would have allowed the collection of more information and the opportunity for more verification of the information that was collected. Second, to reduce the possibility of bias the case studies were carried out by an independent observer. However, this is an expensive process and it is unlikely that programs in LIC will have the resources necessary to commission independent case studies.

Third, more focused research is necessary to assess certain aspects of the programs. For example, client and family satisfaction is an important issue but one that is not easily assessed. Client reports of satisfaction of services may not accurately reflect objective measures of clinical services [24], and, even if measures of client satisfaction do offer valid assessments of the quality of services, conducting such a survey is a major project [25]. Much the same can be said about assessing the ability of livelihood activities to improve the quality of life of clients and families. Revealing why there was such variation in the clinical characteristics of clients in the three programs would also require additional research. Based on the case studies, we can speculate about the reasons: identification and treatment of epilepsy is compatible with other community-based rehabilitation services, the remote location of the Tabaco City program means that only the most serious cases of psychosis and mood disorder will be brought to care, and the ability of the Karakonam program community volunteers to identify persons with common mental disorders is the result of comprehensive case-finding. However, only further research will determine the reasons for the variation. Assessing the effects of Self-Help Groups and Livelihood Programs is another example of research that would add depth to our understanding of community mental health services. Such studies might collect evidence of the effects of these interventions on specific mental disorders by linking data about participation in the Self-Help Groups and/or Livelihood Programs with measures of outcomes and use of services. Qualitative research would be useful, too, because the effects of Self-Help Groups and Livelihood Programs are often found in the domains of personal and family relationships, social reintegration, and feelings of self-worth.

Lack of data was the greatest limitation on our ability to evaluate the effectiveness of interventions. None of the programs had information systems that could easily generate routine process data, and even if process data were available, we believe that clinical and social outcome data are essential for evaluation of program effectiveness [26]. Unfortunately, at the time of the site visits none of the programs routinely assessed and documented the clinical status of clients. To address this deficiency, we are now undertaking a pilot project in Abuja to establish a data collection system that will have the capacity to generate statistics about the socio-demographic and clinical characteristics of clients, their use of services, and measures of clinical, functional, quality of life, and social outcomes. The Karakonam program is now undertaking a similar project.

## Conclusions

We believe that one of the major challenges to scaling-up mental health services is the lack of evidence about the community-based strategies that are effective in delivering efficacious interventions to populations in LIC. The qualitative case study methodology that was used to describe programs in Nigeria, the Philippines, and India begins to address that need by describing service models as they function in practice. The methodology also makes it possible to compare and contrast different models. Finally, case studies such as these generate questions for further investigation and set the stage for more rigorous evaluations of effectiveness [27,28].

## Abbreviations

CBR: Community-Based Rehabilitation; CMD: Common Mental Disorder; CV: Community Volunteer; CPN: Community Psychiatric Nurse; FSG: Family Support Group; FW: Field Worker; NGO: Non-Governmental Organization; SHG: Self-Help Group

## Competing interests

CBM International funded the Case Studies Project, which is lead by Prof Vikram Patel and employs Drs Alex Cohen and Mary De Silva. Drs Julian Eaton and Birgit Radtke are members of the CBM Mental Health Advisory Working Group. Dr Christina George and Bro Victor Manuel lead programs that are supported by CBM International.

## Authors' contributions

AC, VP, MDS, JE, and BR all contributed to the concept and design of the research. AC conducted the site visits, collected data, drafted the manuscript. CG and VM hosted the site visits and reviewed the manuscript for accuracy. All authors reviewed the manuscript and assisted in revising it for important intellectual content. All authors have participated sufficiently in the work to take public responsibility for appropriate portions of the content. All authors have read and approved the final manuscript.

## Authors' information

AC is a Senior Lecturer, MDS is a Lecturer, and VP is a Professor of International Mental Health in the Faculty of Epidemiology and Population Health at the London School of Hygiene & Tropical Medicine. VP is also a Wellcome Trust Senior Research Fellow in Clinical Science. JE is the CBM Mental Health Advisor for West Africa. BR is the CBM Programme Director for Community Mental Health. CG is an Associate Professor, Department of Psychiatry, Dr Somervell Memorial Medical College. VM is the Director of Holy Face Rehabilitation Center for Mental Health.
